# The influence of K-wire transfixation on proximalization of the first metacarpal after resection suspension interposition arthroplasty

**DOI:** 10.1007/s00402-021-03780-9

**Published:** 2021-01-25

**Authors:** Niklas M. Fritz, Ingo Ludolph, Andreas Arkudas, Raymund E. Horch, Aijia Cai

**Affiliations:** Department of Plastic and Hand Surgery, University Hospital Erlangen, Friedrich Alexander University Erlangen-Nuernberg FAU, Krankenhausstrasse 12, 91054 Erlangen, Germany

**Keywords:** CMC1 joint, Osteoarthritis, Resection suspension arthroplasty, Saddle joint, First metacarpal proximalization, K-wire transfixation

## Abstract

**Introduction:**

Osteoarthritis of the first carpometacarpal joint is a common degenerative disease and surgical treatment includes resection suspension interposition arthroplasty (RSIA) with or without temporary transfixation of the first metacarpal. One major drawback includes proximalization of the first metacarpal during the postoperative course. Specific data comparing different transfixation techniques in this context is sparse.

**Materials and methods:**

In this retrospective study, we measured the trapezial space ratio (TSR) in 53 hands before and after RSIA to determine the proximalization of the first metacarpal depending on the type of Kirschner (K)-wire transfixation. We, therefore, compared transfixation of the first metacarpal to the scaphoid with one K-wire (1K) to transfixation of the first metacarpal with two K-wires (2K), either to the carpus (2Ka), or to the second metacarpal (2Kb), or to both second metacarpal and carpus (2Kc).

**Results:**

While preoperative TSR did not differ between group 1K and 2K (*p* = 0.507), postoperative TSR was significantly higher in group 2K compared to 1K (*p* = 0.003). Comparing subgroups, postoperative TSR was significantly higher in group 2Kc than 1K (*p* = 0.046), while we found no significant difference comparing either group 2Ka or 2Kb to 1K (*p* = 0.098; *p* = 0.159). Neither did we find a significant difference within 2K subgroups, comparing group 2Ka and 2Kb (*p* = 0.834), 2Ka and 2Kc (*p* = 0.615), or 2Kb and 2Kc (*p* = 0.555).

**Conclusions:**

The results of our study suggest that transfixation with two K-wires should be preferred to transfixation with one K-wire after RSIA. Specifically, transfixation from first to second metacarpal and from first metacarpal to carpus resulted in least proximalization of the first metacarpal postoperatively.

## Introduction

Osteoarthritis of the first carpometacarpal joint occurs predominantly in women and shows an increasing prevalence with age [1–3]. The disease exhibits an age-adjusted radiographic prevalence of 15% in women and 7% in men 30 years of age or older [2]. Amongst others, biomechanical factors are assumed to contribute to the development of osteoarthritis of the first carpometacarpal joint. Joints with low congruence are susceptible to increased wear of cartilage, which in turn increases the risk of osteoarthritis [4–7]. Additionally, the great range of motion, small contact areas of the joint surfaces and intensive usage exacerbate this issue [8]. In postmenopausal women, a hormonally mediated weakening of the trapeziometacarpal ligaments is discussed, which can additionally lead to decreased joint stability [9]. Obesity and positive family history are considered as risk factors as well [10, 11]. Many patients only suffer from mild symptoms. However, the disease can lead to heavy pain, loss of grip strength and instability of the first carpometacarpal joint in a subset of patients, leading to a pronounced functional impairment of the hand and restrictions in daily routine. During the early stages of the disease, treatment goals are relieving symptoms and slowing the progress while operative procedures are implemented in later stages. Many operative techniques, including ligament reconstruction and/or ligament interposition, have been described by multiple authors [12–15]. A fundamental element of such procedures is the resection of the trapezial bone. Additionally, most techniques create a suspension arthroplasty using either a strip of the flexor carpi radialis tendon or the abductor pollicis longus tendon, with or without interposition to avoid an impingement between the first metacarpal base and the scaphoid [16]. Burton and Pellegrini described a method of postoperative Kirschner (K)-wire transfixation from the first metacarpal to the scaphoid to ensure postoperative stabilization during healing [17], which was also used by others later on [18–20]. However, from a biomechanical point of view, a transfixation of the first metacarpal to the scaphoid seems less suited to limit proximalization of the first metacarpal, as the K-wire lies longitudinally to the direction of muscle traction. Thus, a more horizontal vector of transfixation would be more appropriate.

Data on the actual shrinkage of the resection cavity of the trapezium are sparse and includes only small case numbers [17, 21].

To evaluate the influence of temporary K-wire transfixation on the proximalization of the first metacarpal bone, we retrospectively analyzed different methods of K-wire transfixation during resection suspension interposition arthroplasty (RSIA) by radiographic means.

## Materials and methods

### Collective

**Fig. 1:**
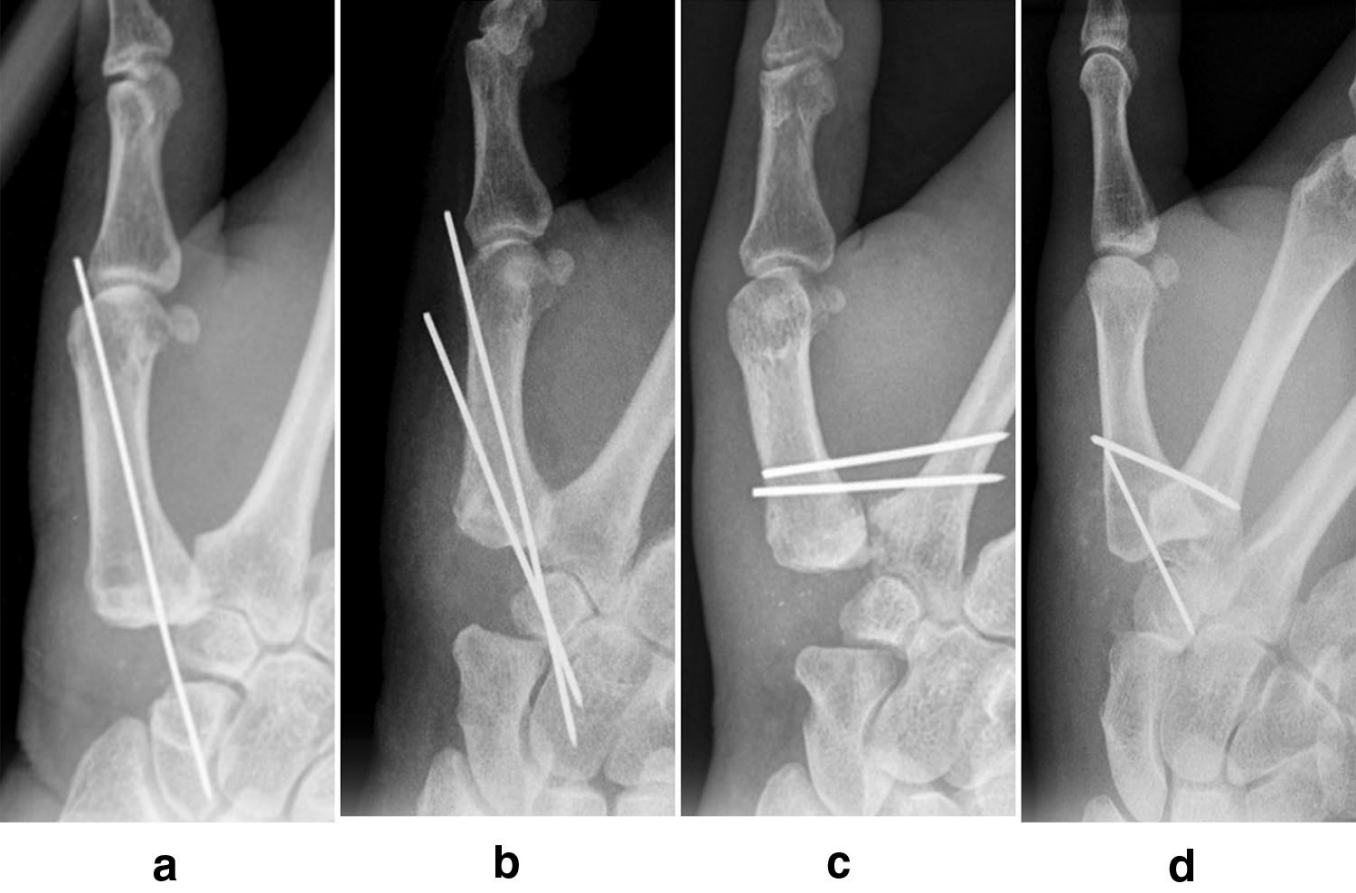
Methods of transfixation. Patients received transfixation with one K-wire from the first metacarpal to the scaphoid (group 1K, **a**), or with two K-wires from the first metacarpal to the carpus (group 2Ka, **b**), from the first to second metacarpal (group 2Kb, **c**) or from the first to both second metacarpal and carpus (group 2Kc, **d**)

All patients having undergone RSIA in our clinic for osteoarthritis of the first carpometacarpal joint between 2005 and 2019 were retrospectively analyzed. For analysis, pre- and postoperative radiographs of the thumb were necessary. Thus, patients without adequate radiographs or follow-up were excluded. Of the remaining cases, those were included which had either received K-wire transfixation of the first metacarpal base to the scaphoid (group 1K, Fig. 1a), or two K-wires for transfixation of the first metacarpal (group 2K). Group 2K was further divided into subgroups according to the manner of transfixation: transfixation of the first metacarpal to the carpus (group 2Ka, Fig. 1b), to the second metacarpal (group 2Kb, Fig. 1c) or to both carpus and second metacarpal (group 2Kc, Fig. 1d).

To evaluate the influence of K-wire transfixation on proximalization of the first metacarpal, the trapezial space ratio (TSR) was measured for each group and subgroup via preoperative as well as postoperative radiographs as described below.

### Calculation of the TSR

**Fig. 2:**
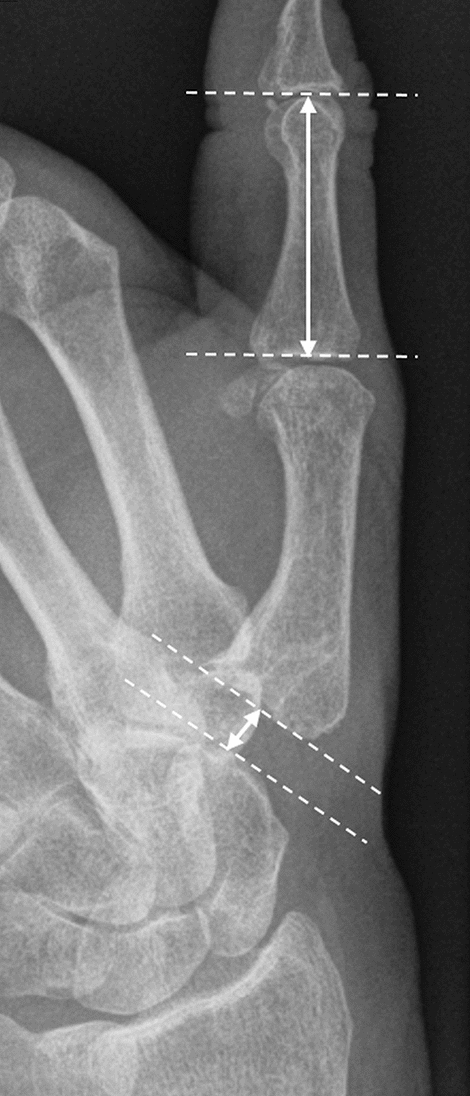
Calculation of the TSR. The TSR is calculated as the fraction of height of the trapezial space and the length of the proximal phalanx of the thumb

The TSR is a measurement of the space occupied by the trapezial bone and was calculated as described by Kadiyala et al. [22]. Conventional X-ray images of the affected thumb were taken. The distance between the subchondral surfaces of the first metacarpal base and the distal scaphoid was measured. Likewise, the length of the proximal phalanx of the thumb was measured between the subchondral surface of the base and the distal aspect of the first proximal phalanx (Fig. 2) and rounded to 0.1 mm. The TSR was calculated as the ratio of these two measurements and was rounded to three decimal places.

### Operating technique and follow-up treatment

RSIA was performed as described by Beckenbaugh-Linscheid [23] by different senior physicians in our clinic. Mainly, a radiographic stage three of the first CMC osteoarthritis after Eaton and Littler was found preoperatively (Table 1). The stage had no influence on the manner of transfixation. Patients both with and without thumb adduction were included in the analysis. At first, the trapezial bone is resected via a dorsal incision [24]. Then a strip of about one-third of the flexor carpi radialis tendon is prepared, leaving it attached distally. Then it is pulled through a hole drilled through the base of the first metacarpal, led back to the tendon insertion and sutured to itself. This results in the first metacarpal base being suspended from the second metacarpal base. The rest of the strip of the flexor carpi radialis tendon is used as a spacer for the resected trapezial bone. At the end of the operation, K-wire transfixation of the first metacarpal base was performed during distraction of the thumb ray as described above. For transfixation, K-wires of 1.2 mm ± 0.1 mm were used. Time of K-wire removal was depicted from patient records. Immobilization of the thumb took place in a cast or an orthosis for 5 weeks postoperatively. After immobilization, the patients received physiotherapy, increasing the load on the hand gradually.

**Table 1:** Biometrical data and radiographic stages of first CMC osteoarthritis in collective

Group	Number of female patients	Number of male patients	Mean age (years)	Radiographic stage of first CMC osteoarthritis after Eaton and Littler			
				*I*	*II*	*III*	*IV*
1K	16	4	68.0	–	4	12	5
2Ka	9	–	56.3	–	2	5	2
2Kb	6	2	57.1	–	2	4	2
2Kc	13	2	60.0	–	2	9	4

### Statistical analysis

GraphPad Prism 7.0 (GraphPad Software Inc., La Jolla, CA, USA) was used for statistical analysis. Descriptive statistics were expressed as mean and standard deviation. Shapiro–Wilk test was used to assess data normality. An unpaired *t* test or Mann–Whitney test was used, as appropriate. Significance was set at a *p* value < 0.05.

## Results

Between 2005 and 2019, there were 117 RSIA performed in a total of 107 patients. There were 10 patients, who had received RSIA on both hands. 59 cases with no adequate postoperative radiograph or follow-up were excluded from the study. Of the remaining 58 cases, 21 cases were included with one K-wire, transfixating the first metacarpal base to the scaphoid (group 1K). There was one case with one K-wire transfixation from the first to second metacarpal and four cases with one K-wire transfixating the first metacarpal to the trapezoid bone. Those two techniques of K-wire transfixation were not included in our analysis due to the small number of cases. There were 32 cases with two K-wires in total (group 2K). Of those 32 cases, there were 9 cases in group 2Ka (first metacarpal to carpus), 8 cases in group 2Kb (first to second metacarpal), and 15 cases in group 2Kc (first metacarpal to both carpus and second metacarpal). In group 1K, we found a gender distribution of 16 women to four men (one woman having had received RSIA on both hands), in group 2Ka of 9 women only, in group 2Kb of 6 women to 2 men and in group 2Kc of 13 women to 2 men (Table 1). The average age in the collective was 62 years at the time of the operation. No revision surgeries were necessary. K-wire removal took place after 13.6 ± 8.1 days on average. Follow-up radiographs were obtained 257 ± 251 days postoperatively on average. Follow-up did not differ much comparing group 1 vs. 2K (286 days ± 297 vs. 250 days ± 221).

**Fig. 3:**
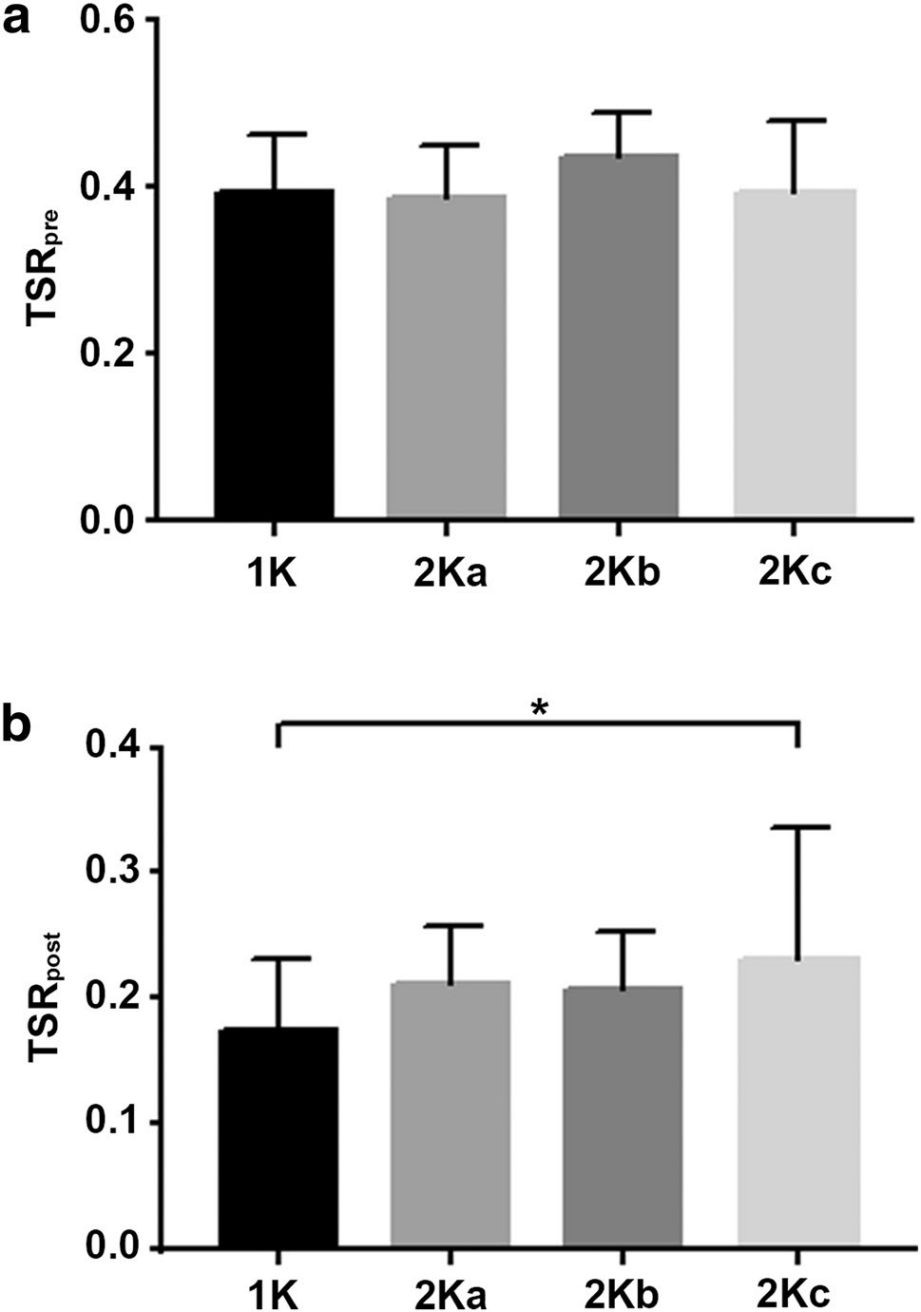
**a** Preoperative TSR-values. Preoperative TSR-values (TSR_pre_) did not differ between group 1K and 2Ka, b or c. We found no significant difference between 2Ka and 2Kc (*p* = 0.414), 2Kb and 2Kc (*p* = 0.215) or 2Ka and 2Kb (*p* = 0.139). Statistical significance between groups was tested with two-tailed unpaired *t* test or two-tailed Mann–Whitney test as appropriate. *p < 0.05. **b** Postoperative TSR-values. Postoperative TSR-values (TSR _post_) were significantly higher in group 2Kc compared to 1K (*p* = 0.046. No significant difference was found when comparing group 2Ka or 2Kb to 1K (*p* = 0.098, *p* = 0.159, respectively) and within 2K subgroups [*p *(2Ka vs. 2Kb) = 0.843, *p* (2Ka vs. 2Kc) = 0.615, *p *(2Kb vs. 2Kc) = 0.555]. Statistical significance between groups was tested with two-tailed unpaired *t* test. *p < 0.05

Preoperatively, we observed an average TSR of 0.392 ± 0.069 in group 1K. Postoperatively, the TSR decreased to 0.174 ± 0.058 in this group. In group 2K, there was a preoperative TSR of 0.402 ± 0.073, which decreased to 0.22 ± 0,078 after surgery. When analyzing 2K subgroups, a decrease from a preoperative TSR of 0.386 ± 0.065 to a postoperative TSR of 0.212 ± 0.047 for group 2Ka could be shown. A similar trend was observed for group 2Kb (preoperative TSR = 0.436 ± 0.051, postoperative TSR = 0.207 ± 0.045) and group 2Kc (preoperative TSR = 0.394 ± 0.085, postoperative TSR = 0.231 ± 0.105). Statistical analysis revealed no difference between the preoperative TSR of group 1K and 2K (*p* = 0.507) as well as between the preoperative TSR of 1K and 2K subgroups (1K vs. 2Ka, *p* = 0.829, 1K vs. 2Kb: *p* = 0.139, 2Kc: *p* = 0.958). Within 2K subgroups, there was also no significant difference between preoperative TSR (2Ka vs. 2Kb: *p* = 0.139, 2Ka vs. 2Kc: *p* = 0.414, 2Kb vs. 2Kc: *p* = 0.215) (Fig. 3a). Postoperative TSR was significantly higher in group 2K compared to 1K (*p* = 0.003). Additionally, subgroup 2Kc showed a higher postoperative TSR value compared to 1K (*p* = 0.046). No difference could be shown between group 1K and 2Ka (*p* = 0.098) or 1K and 2Kb (*p* = 0.159), however, with a trend towards higher TSR values in 2K subgroups without reaching statistical significance with respect to group 1K. Again, postoperative TSR values within 2K subgroups did not differ (2Ka vs. 2Kb: *p* = 0.843, 2Ka vs. 2Kc: *p* = 0.615, 2Kb vs. 2Kc: *p* = 0.555) (Fig. 3b).

## Discussion

Due to its extraordinary anatomy, the first carpometacarpal joint with its high degree of thumb movement is especially susceptible to osteoarthritic changes [8]. Long-term follow-up studies after surgical therapy of symptomatic advanced osteoarthritis of the first carpometacarpal joint through RSIA show largely positive subjective and objective treatment outcomes [25–30]. An obvious and generally acknowledged problem is that the distance between the first metacarpal base and the scaphoid decreases in the long term after resection of the trapezial bone. This can lead to an impingement with corresponding clinical symptoms [31, 32]. Furthermore, a recent study showed the importance of maintaining thumb length due to an inverse correlation of thumb length with overall Disabilities of the Arm, Shoulder, and Hand (DASH) scores and specific DASH questions involving high-strength activities [33]. To address this problem, different concepts with ligament reconstruction [34], tendon interposition [25], joint replacement [35–37], or spacers have been proposed [38, 39]. However, the earlier propagated use of silicon interposition resulted in rapid abrasion and fast reduction of the resection cavity [40–42], which is why larger registers dissuade their use [43].

Kadiyala et al. examined 15 patients with symptomatic degenerative first carpometacarpal osteoarthritis before and after surgical treatment with ligament reconstruction and tendon interposition arthroplasty. They found a TSR of 0.372 ± 0.084 in preoperative and of 0.270 ± 0.078 in postoperative X-rays of thumbs with symptomatic first carpometacarpal osteoarthritis [22]. Having found a TSR reduction of 43% in comparison to healthy thumbs, they emphasized the importance of ligament reconstruction and tendon interposition for maintaining the length of the thumb, which is essential for grip function [44]. In 1986, Burton and Pellegrini described excellent postoperative results after RSIA and transfixation of the first metacarpal to the scaphoid for osteoarthritis of the first carpometacarpal joint. They described that proximalization of the first metacarpal averaged only 11% of the initial arthroplasty space versus nearly 50% loss of height with silicone implants [17]. However, the role of K-wire transfixation in preventing proximalization is not clear yet, as multiple studies have shown no clinical benefit after ligament reconstruction and transfixation compared to trapeziectomy alone [19].

To the best of our knowledge, this is the first study to correlate proximalization of the first metacarpal to the manner of K-wire transfixation after RSIA. We were able to show that transfixation with two K-wires seems to be superior to transfixation with one K-wire when it comes to preventing proximalization of the first metacarpal (represented by higher TSR values) in the postoperative course. However, when analyzing 2K subgroups, we only found a statistical difference between group 2Kc and 1K, although there was a trend towards higher postoperative TSR values in group 2Ka and 2Kb compared to 1K.

This indicates an important role of transfixating the first metacarpal to two different structures, namely second metacarpal and carpus. This results in two different vectors of transfixation, thus preventing subsidence in either direction during the postoperative healing process by a “blocking” function of each K-wire.

One limitation of this study is its retrospective character, leading to only a small number of included cases in spite of the large number of RSIA performed at our institution. Some patients were lost to follow up and in other cases, no adequate radiographic examination was available. Furthermore, the technique of K-wire transfixation was heterogeneous, leading to several subgroups with exclusion of some subgroups due to the small number of cases. We want to emphasize that the presented data cannot give a direct impression of the clinical impairment of the patients [21], as this is solely a morphologic study. The examination of x-rays without a survey of clinical or subjective data can naturally not compare pre- and postoperative symptom scores, like the DASH score. However, we know from previous research, that loss of trapezial hight correlates inversely with clinical outcome [33], making it necessary to find ways to limit proximalization of the first metacarpal.

Additionally, it seems advisable to ensure a position of the first metacarpal as distal as possible to maintain physiological anatomical conditions from a biomechanical point of view. This can both prevent painful impingement between the first metacarpal base and the scaphoid and on the other side enables a physiological direction of pull and force development of the involved extrinsic and intrinsic thumb muscles. We conclude based on measurements of the radiographic morphology, that an anatomical reconstruction of the first metacarpal position should be valued highly to avoid potential disadvantages for the patient. Upon deciding to stabilize the first metacarpal base postoperatively, a preferably rigid manner of K-wire transfixation should be chosen to maintain a maximal distance between the first metacarpal base and the scaphoid in the long term. Thus, we propose to transfixate the first metacarpal to the second metacarpal as well as to the carpus after RSIA.
